# Two Congenital Gastrointestinal Malformations: A Case of Omphalocele and Meckel’s Diverticulum in a Neonate

**DOI:** 10.7759/cureus.62603

**Published:** 2024-06-18

**Authors:** Débora Valente, André Assunção, Carolina Aquino, Cristina Ferreras, Filipa Flor-de-Lima, Susana Pissarra

**Affiliations:** 1 Pediatrics, Centro Hospitalar Universitário de São João, Porto, PRT; 2 Pediatric Surgery, Centro Hospitalar Universitário São João, Porto, PRT; 3 Neonatology, São João University Hospital Center, Porto, PRT; 4 Neonatology, Centro Hospitalar Universitário São João, Porto, PRT; 5 Neonatology, Faculty of Medicine of Porto University, Porto, PRT; 6 Neonatal Intensive Care Unit, Centro Hospitalar Universitário de São João, Porto, PRT

**Keywords:** pediatric surgery, pediatrics & neonatology, neonate, meckel´s diverticulum, omphalocele

## Abstract

Omphalocele is a malformation of the abdominal wall varying in size and located at the base of the umbilical cord. Meckel's diverticulum is the most common congenital malformation of the gastrointestinal tract with an increased prevalence in newborns with congenital malformations of the umbilicus and gastrointestinal tract. The association between Meckel's diverticulum and omphalocele has been described in rare cases. We present the case of a newborn diagnosed with both entities.

## Introduction

Omphalocele is a malformation of the abdominal wall, of variable size at the base of the umbilical cord [1]. The literature distinguishes omphaloceles as major and minor (minor <4 cm and major >4 cm) [2] with smaller defects typically resolving within the first 24 to 72 hours [1]. Its estimated prevalence is approximately 2.6 per 10,000 births [1], and it is often associated with multiple malformation syndromes.

Meckel's diverticulum is the most common congenital malformation of the gastrointestinal tract, with its prevalence increased in newborns with congenital malformations of the umbilicus and gastrointestinal tract [3,4]. The association between Meckel's diverticulum and omphalocele has been described as having a higher incidence in cases of minor omphalocele, but its true incidence is unknown.

In this context, we present the case of a newborn diagnosed with both entities [5-7].

## Case presentation

**Figure 1:**
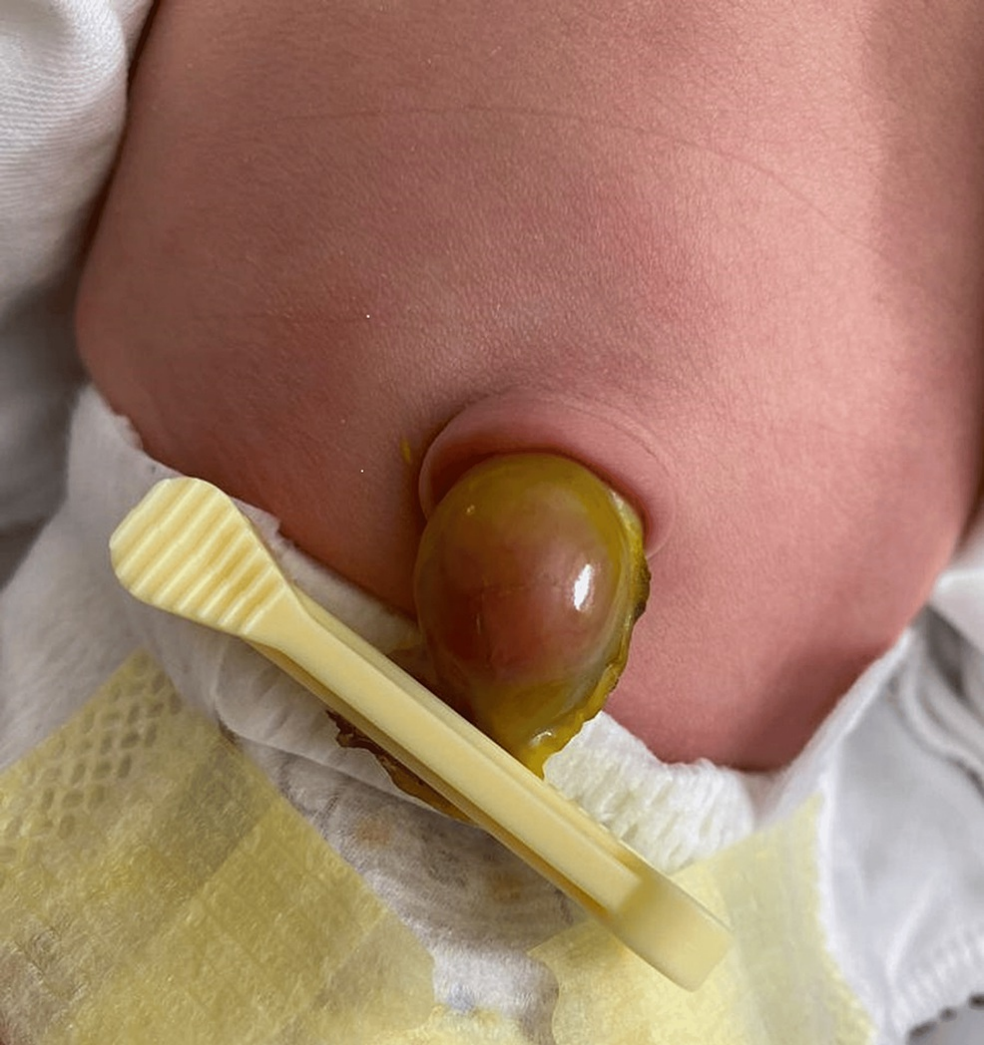
Omphalocele at presentation

A newborn at term, from a monitored and uneventful pregnancy, was transferred on the second day of life due to a suspected incarcerated umbilical hernia. Upon admission, a voluminous umbilical stump was observed (Figure 1).

Also noted was the presence of syndactyly of the second and third toes bilaterally. Due to suspected intestinal obstruction, an ultrasound was performed confirming the presence of a herniation with enteric content and an anteroposterior diameter/caliber of the hernial neck of 6-7 mm without obstruction. A pediatric surgical consultation was also requested, detecting a defect covered by an intact membrane (minor omphalocele), containing a structure suggestive of an intestinal loop. An unsuccessful attempt at reduction was made. Due to the onset of excoriation and the inability to reduce the hernia conservatively, surgery was scheduled.

**Figure 2:**
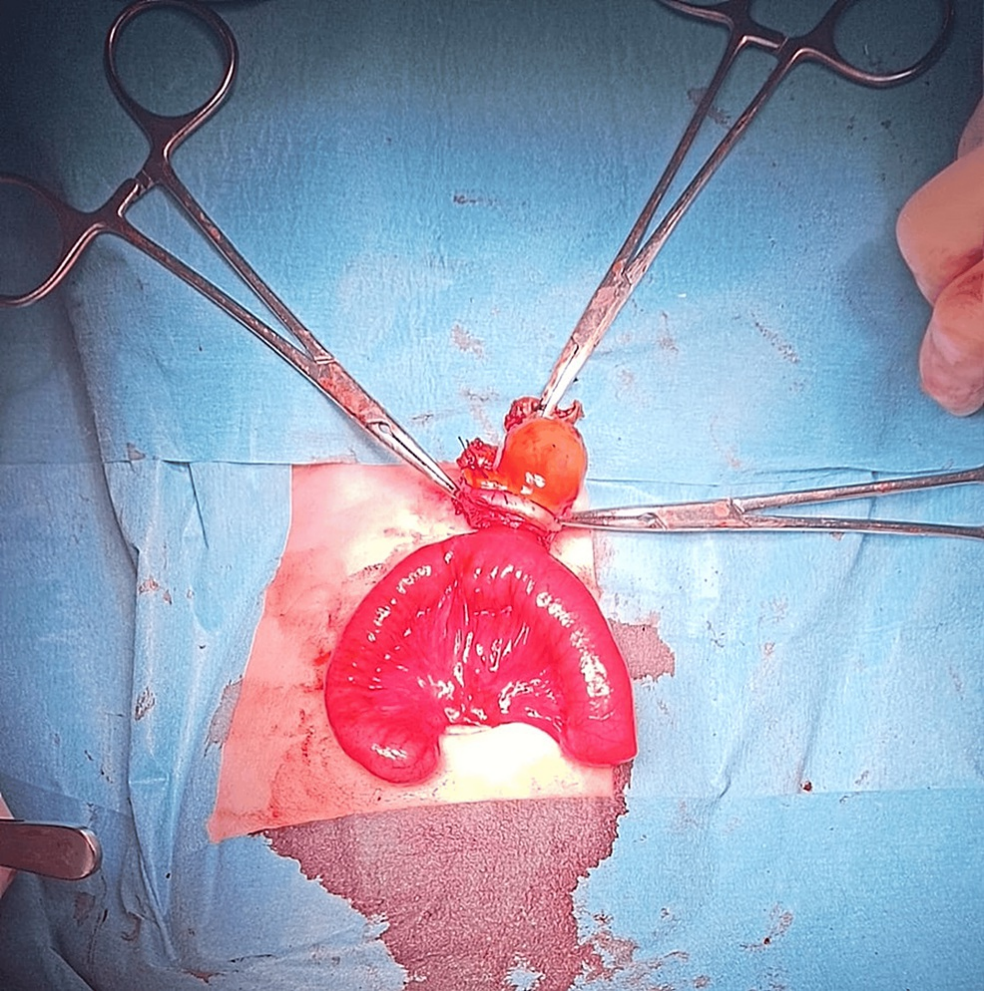
Meckel’s diverticulum isolated during surgery

On the fourth day of life, the newborn underwent surgical correction of the defect. During the surgery, the presence of omphalocele was confirmed. Also, a Meckel's diverticulum contained within the omphalocele was isolated (Figure 2) leading to a decision to perform a segmental enterectomy followed by the reduction of the intestinal contents and closure of the defect. Clinical evolution was favorable.

## Discussion

Omphalocele is a malformation of the abdominal wall, with an estimated prevalence of 2.6 per 10,000 births, covered by a membranous sac [1]. The umbilical cord inserts into the apex of this sac, typically containing herniated abdominal contents. The pathogenesis of this condition is not fully understood, although various mechanisms have been proposed.

During the fourth and fifth weeks of gestation, the embryonic disc folds in four different directions, with each fold converging at the location of the umbilicus. By the sixth week of gestation, the abdominal cavity becomes too small to accommodate all its contents, leading to the protrusion of the midgut, forming a temporary hernia that wraps 90 degrees around the superior mesenteric pedicle, called the physiological hernia of the midgut [1,8,9]. Subsequently, reduction of the hernia involves an additional 270-degree rotation within the abdominal cavity and typically occurs by the twelfth week of gestation, rendering the hernia physiological from this point onward [8,9].

Omphalocele is often associated with other congenital malformations, with frequencies varying between 27% and 63%, affecting the heart, musculoskeletal system, urogenital system, digestive system, lungs, and central nervous system [2,10-12].

Meckel's diverticulum results from incomplete obliteration of the vitelline duct, leading to its formation. The omphalomesenteric duct typically involutes between the fifth and sixth weeks of gestation, while the intestine establishes its definitive position in the abdominal cavity. The persistence of this duct can result in various anatomical alterations, one of which is Meckel's diverticulum. As described in this case, the diverticulum may be attached to the omphalocele sac, which may contribute to the intestine not returning to the abdominal cavity during gestation [3,8,9].

The concurrent incidence of omphalocele and Meckel's diverticulum is not well known but is uncommon, with studies reporting its occurrence in 16% of omphalocele cases [4-6].

Treatment of this condition involves surgical resection of the intestinal portion comprising the diverticulum and subsequent intestinal anastomosis. Correction of the abdominal wall is performed afterward [8].

In this case, due to the small size of the omphalocele, both corrections were possible during the same surgical procedure. This facilitated a rapid and favorable outcome for the newborn, who is currently asymptomatic and under follow-up in the clinic. During this follow-up and given the detected alterations (syndactyly), genetic testing is being conducted to identify possible alterations that may predispose to these abnormalities, as the presence of omphalocele is associated with various genetic syndromes.

## Conclusions

The concurrent presence of a Meckel's diverticulum and an omphalocele is not common but has been described. Surgical resection and repair of the abdominal wall defect are the first-line treatment, typically resulting in a favorable outcome. Although an umbilical cord hernia was initially considered, we were dealing with a case of omphalocele.

With this case, the authors aimed to draw attention to the careful evaluation of the umbilical cord. When faced with a voluminous umbilical cord, it is important to consider the possibility of an omphalocele and investigate it thoroughly.
